# Clinical Characteristics and Etiology of Bilateral Vestibular Loss in a Cohort from Central Illinois

**DOI:** 10.3389/fneur.2018.00046

**Published:** 2018-03-01

**Authors:** Jorge C. Kattah

**Affiliations:** ^1^Illinois Neurologic Institute, University of Illinois College of Medicine, Peoria, IL, United States

**Keywords:** bilateral vestibulopathy, acute bilateral vestibular loss, chronic bilateral, vestibular loss, neurologic disorders associated with bilateral vestibular loss

## Abstract

**Background:**

Previous series of bilateral vestibular loss (BVL) identified numerous etiologies, but surprisingly, a cause in a significant number of cases remains unknown. In an effort to understand possible etiology and management strategies, a global effort is currently in progress. Here, I contribute my 10-year experience with both acute and chronic BVL during the 2007–2017 decade.

**Methods:**

This is a retrospective review of the charts and EMR of patients diagnosed with BVL in the last 10 years. Following Institutional IRB approval, we identified 57 patients with a diagnosis of BVL and utilized the current diagnostic criteria listed by the Barany society (1). The inclusion criteria included patients with BVL of any cause, within an age span older than 18 and a neuro-otologic examination supporting the clinical impression of BVL.

**Results:**

During the current decade 2007–2017, I identified two broad categories of BVL (acute and chronic) in 57 patients; only 41 of them had records available. The etiology includes: idiopathic: *n* = 9, Wernicke’s encephalopathy *n* = 11, superficial siderosis *n* = 3, paraneoplastic syndrome: *n* = 3, bilateral vestibular neuritis (recurrent AVS lasting days without cochlear symptoms) *n* = 3, simultaneous ototoxicity of aminoglycoside and chemotherapy toxicity *n* = 2, MELAS *n* = 2, Meniere’s disease treated with intra-tympanic streptomycin in one ear *n* = 1, acute phenytoin intoxication: *n* = 1, combined chronic unilateral tumor-related vestibulopathy and new contralateral vestibular neuritis (this patient presented with Betcherew’s phenomenon) *n* = 1, bilateral AICA stroke *n* = 1, mixed spinocerebellar ataxia type 3, *n* = 2 and CANVAS *n* = 2.

**Conclusion:**

This cohort included a 28% overall incidence of acute and subacute BVL; among them, 65% improved with intervention. In the thiamine deficiency group, specifically, the vestibular function improved in 80% of the patients. Even though acute, subacute, or chronic showed slightly asymmetric horizontal-VOR gain loss, it never did cause spontaneous, primary straight gaze horizontal nystagmus. *n* = 39/41 patients had abnormal manual HIT, *n* = 26/41 BVL patients tested with video head impulse immediately after manual testing showed decreased VOR gain, including two with covert saccades. Two thiamine patients with positive bedside pretreatment manual HIT, tested after treatment with high-dose thiamine showed improved VOR. In acute thiamine deficiency, the horizontal VOR was abnormal and the vertical was either normal or mildly decreased. This series favored a neurologic cause of BVL. Finally, 20% of the chronic cases were idiopathic.

## Introduction

Bilateral vestibular loss is uncommon with an estimated incidence of 28/100,000 (1); however, despite advances in early diagnosis, and significant progress in vestibular testing, and imaging, parallel to molecular screening for genetic and immune BVL phenotypes, a significant number of patients remain idiopathic (2–4). One important and potentially preventable cause of BVL is exposure to ototoxic drugs with typical subacute onset of symptoms. Awareness of potential ototoxicity from antibiotics and close monitoring of patients with infections without other viable treatment led to preventive clinical measures and the use of serum levels to identify an early threshold of ototoxicity (5). In addition, I identified in these cohort patients with acute BVL due to thiamine deficiency and other less common causes. This group displayed overlapping symptoms and signs with the BVL but had acute onset, affected a specific group individuals at risk of nutritional deficiency and had additional clinical findings; timely recognition of this syndrome may lead to recovery. Although awareness of these higher risk patients is greater than 40 years (6–8). In this retrospective study, we analyze their clinical characteristics as a group (9–11). Here, I report my experience with 41 acute and subacute/chronic BVL patients studied during the past decade.

## Materials and Methods

The University of Illinois College of Medicine IRB approved this study. We report the result of a retrospective chart review of patients with BVL diagnosed between years 2007 and 2017, 57 patients had a final diagnosis of BVL at our Center. Here, I classified this cohort according to the duration of the symptoms, the first group comprised either acute (less than 24-h), or subacute (less than 1 month) duration; and a second group with long-standing symptoms (greater than 1 month), I utilized the recently published diagnostic criteria from the Barany Society (12); only 41 of them had records available. Inclusion criteria included comparatively better static than dynamic visual acuity (VA) (more than 3-line deterioration), truncal imbalance, positive manual head impulse test in horizontal canal planes, and in some cases, positive vertical canal compromise, in these cases, we confirmed decreased VOR gain (below: 0.7). Bilaterally reduced caloric response with maximum slow phase velocity of <5°/s and torsional chair with decreased gain (<0.6), phase lead greater than 68° and bilaterally low time constants of the step rotational induced nystagmus (<5 s). All patients underwent a neurologic and vestibular clinical evaluation that included a mental status assessment to assess orientation and determine level of awareness and ability to follow the examination protocol.

Patients with a history of alcohol dependence or nutritional deficiency underwent a specific protocol. The acute cases had a vestibular and ocular motor examination at bedside including manual and video head impulse (vHIT) followed by a neurologic examination. I repeated these tests after the intravenous administration of 500 mg of thiamine intravenously (9, 11). I ordered MRI of the brain in all cases. In patients with a subacute history, we measured serum thiamine levels and began oral thiamine replacement.

When the history suggested BVL, I implemented the following protocol: static followed by dynamic VA, direct ophthalmoscopy, visual field testing, oculomotor and cranial nerve testing, nystagmus analysis if present (both with fixation and with fixation block), manual head impulse in all six-canal planes. Truncal posture including Romberg test with eyes open and closed and balance while standing on a foam cushion. I tested gait and tandem gait whenever possible. Acute patients unable to stand had their ability to sit at the side of the bed with the arms crossed and eyes closed. At this point, I tested fine motor limb coordination, muscle tone, strength, and pathologic reflexes. We concluded the examination with nystagmus provocative maneuvers (routinely hyperventilation, mastoid vibration, Valsalva maneuver, horizontal head shaking, and positional testing). Simultaneous visual vestibulo-ocular reflex (VVOR) testing was performed only in our most recent cases.

If the clinical assessment suggested BVL, the work-up ordered included pure tone audiometry and vestibular testing including: Nystagmus recordings, bithermal caloric stimulation, torsion swing chair testing, and the vHIT; I did not order additional otolith function tests. At least one test of vestibular function in each case. Ideally, bithermal caloric testing; the vHIT measure different vestibular receptor frequencies and thus preferably in all patients, particularly if Meniere’s disease is suspected (4, 13, 14). VOR testing with the torsion/swing chair was performed in a few patients as well; however, several patients in this cohort underwent only one test of vestibular function, due to cost or availability issues. After 2012, all patients with BVL had manual and video HIT. With two exceptions, I tested acute cases at the bedside. MRI of the cerebellopontine angle included pre- and post-contrast images. Temporal bone CT scan with if necessary and routine blood tests to screen for metabolic abnormalities, vasculitis, autoimmune disorders, syphilis and Lyme’s serology, thiamine, folate and vitamin B12 serum levels and cerebrospinal fluid studies in selective patients with BVL and additional neurologic abnormalities. In addition, patients with BVL and additional neurologic abnormalities underwent testing in search of an autoimmune and paraneoplastic ataxia.

Follow-up longer than 1 year or greater was accomplished in *n* = 31/41 patients, some have been followed for ~10 years; this follow-up allowed us to assess the clinical course and the effect of medical treatment if any and assessment of the contribution of a defined Physical Therapy rehabilitation protocol.

## Results

I made a diagnosis of BVL in 57 patients at our Center; only 41 of them had records available (Tables 1 and 2). The average age was 58, with a range of 19–86; the gender distribution was 21 females and 20 males. The clinical course was characterized by a slowly progressive bilateral isolated vestibular or cochleovestibular loss in 29 patients and an acute vestibular syndrome in eight and acute sequential in four patients. BVL was associated with simultaneous CNS compromise in 28 and was isolated vestibular or cochleovestibular loss in 13 patients. Bilateral mixed cochleovestibular loss was present in three patients with superficial siderosis and two with MELAS, unilateral postsurgical deafness (unilateral jugular paraganglioma resection), and unilateral Meniere’s (post-unilateral transtympanic streptomycin injection).

**Table 1:** Acute and subacute bilateral vestibulopathy.

Patient	Age	Vestibular test	Classification	Etiology and CNS clinical imaging findings	Video head impulse (vHIT)	Previous vertigo attacks	Outcome over time
1	53	Manual HIT+ Absent Calorics	Subacute	Alcoholism Vitamin B1 deficiency Gaze evoked nystagmus (GEN) DBN Ataxia Vermis Atrophy	RH 0.72^a^ LH 0. 75 RA 0. 76 LA: 0.41 RP: 0.51 LP: 0.76	No	Partial Improvement Truncal Ataxia DBN
2	50	Horizontal Manual HIT+ Vertical Normal Manual HIT Absent Calorics	Acute Vestibular Syndrome	Alcoholism Vitamin B1 deficiency GEN	Not performed	No	Improved
3	55	Manual HIT+Absent Calorics	Subacute	Alcoholism Vitamin B1 deficiency GEN	Not Performed	No	Improved
4	37	Manual HIT+	Subacute	s/p Gastric Bypass Vitamin B1 deficiency GEN/UBN	Not performed	No	Improved
5	39	Manual HIT+ Absent Calorics No ice response	Acute	s/p Gastric Bypass Vitamin B1 deficiency Abducens Paresis GEN	Not performed	No	Improved
6	60	Manual HIT+ Absent Calorics No ice response	Subacute	s/p gastric Bypass Vitamin B1 deficiency Abducens Paresis GEN	RH: 0.50 LH 0.40	No	Improved
7	28	Manual HIT+ vHIT+	Subacute	s/p gastric Bypass Vitamin B1 deficiency	RH: 0.58 LH: 0.64	No	Improved
8	45	Manual HIT+ vHIT+	Acute	Alcoholism Vitamin B1 Deficiency Signal Changes in the medial Vestibular Nuclei and CC Marchiafava Bignami encephalopathy UBN	RH 0.51 LH 0.60 RA 0.93 RP 0.78 LA 0.51 LP 095	No	Improved
9	45	Manual HIT+ vHIT+ (normal months later)	Acute	Alcoholism Vitamin B1 Deficiency Encephalopathy UBN Transition DBN	RH: 0.75^a^ LH: 0.83 LA: 0.65 RP: 0.82 RA: 0.73 LA: 0.75	No	Partial Improvement Truncal Ataxia DBN
10	60	Manual HIT+ vHIT+	Acute	Alcoholism Vitamin B1 Deficiency Encephalopathy UBN Transition DBN MRI Signal changes in midbrain, pons and medulla	RH 0.34 LH 0.39 LA 0.80 RP 0.17 RA 0.15 LP 0.20	No	No Improvement Permanent Low VOR gain Truncal Ataxia DBN Encephalopathy Improved
11	22	Manual HIT+ vHIT+	Acute Encephalopathy With UBN Transition To DBN	s/p gastric Bypass Vitamin B1 Deficiency	RH 0.62 LH 0.57 LA 0.82 RA 0.62 LP 0.69 RP 0.60	No	No Improvement Permanent Low VOR gain Truncal Ataxia DBN Encephalopathy Improved
12	63	Manual HIT+ vHIT+	Acute phenytoin (level: 26.6)	Phenytoin Overdose Ataxia GEN	RH 0.38 LH 0. 43 RA 0.67 LA Not done RP Not done LA 0.27	No	Improved
13	67	Manual HIT+ vHIT+	Acute Rapidly progressive	Anti-Yo PNS Cerebellar Cancer Esophagus DBN Ataxia	RH 0.57 LH: 0.89	No	Progressive course Despite PLEX Chemotherapy steroids
14	62	Manual HIT+ vHIT+	Acute	Anti-Hu PNS Prostate Neuro-endocrine Syndrome Eventually INO = UBN	RH: 0.42 LH: 0.31 RA: 0.15 LP: 0.22 RP: 0.15 LA: 0.17	No	Progressive course Despite PLEX Chemotherapy steroids
15	64	Manual HIT+ vHIT+	Slowly Progressive Sudden worsening	PNS? Ataxia Neuropathy + P/Q Channel antibodies Old ovarian ca	RH 0.53 LH 0.44 RA 0.74 La 0.03 RP 0.46 LP 0.85	No	Did not want immuno-suppression or PLEX
16^a^	63	Manual HIT + vHIT done ~ after new VN	Chronic unilateral Acute Contralateral	Chronic left Vestibular loss Acute right vestibular neuritis	RH 0.86^b^ LH: 0.58 RA 0.45 LA 0 75 RP 0 66 L 0 22	No	Improved Spontaneously. Had Betcherew’s Phenomenon

*^a^vHIT performed 2 weeks after treatment initiation*.

*^b^vHIT performed after discharge when she improved*.

*VN, vestibular neuritis*.

**Table 2:** Chronic bilateral vestibulopathy.

Patient	Age	Vestibular test	Classification	Etiology and CNS clinical imaging findings	Video head impulse (vHIT) gain	Previous vertigo events	Audiometry
1	64	Manual and vHIT+ Torsion swing Abnormal Calorics: depressed	Recurrent Vertigo Bilateral vestibular loss (BVL)	Idiopathic Sjogren’s	RH 0.53 LH 0.39 RA 0.27 LA 0.40 RP 0 12 LP 0 01		Normal
2	78	Manual and vHIT+ All canals	Slowly Progressive BVL	Ataxia MRI Superficial siderosis	Not done	No	Bilateral deafness
3	19	Manual and vHIT+ Torsion swing Calorics: No ice response	Slowly Progressive BVL	Idiopathic History Of migraine	RH 0.62 LH 0.77 RA 0.32 LA 0.58 RP 0 54 LP 0.62		Normal
4	62	Manual and vHIT+	Recurrent Vertigo BVL	Idiopathic	RH 0.14 LH 0.47 RA 0.39 LA 0.51 RP 0.28 LP 0.40	Yes	Normal
5	73	vHIT Calorics Depressed Max slow phase velocities (SPV)* 4°/s	Slowly Progressive BVL	CANVAS DBN Ataxia neuropathy	RH 035 LH 0 28 RA 069 LA 0.36 RP 0 36 LP 0 35	No	Normal
6	57	Manual and vHIT+	Slowly Progressive BVL	Presumed Siderosis Old SA Hemorrhage DBN Ataxia	RH 0 55 LH 0 27 RA 030 LA 0 56 RP 0 47 LP 0 91	No	60 dB loss R > L
7	54	Manual and vHIT+	Slowly Progressive BVL	Gentamicin and Carbo-Platinum For Uterine Cervix cancer	RH 0 01 LH 0 02 RA 0 23 LA 0 10 RP 0 14 LP 0 19	No	
8	39	Manual and vHIT+	Slowly Progressive BVL	Gentamicin Ototoxicity	RH 0 59 LH 0.64 RA 0 37 LA 0.61 RP 0 50 LP 0 18	No	Mixed hearing Loss R ear High frequency Hearing Loss L ear
9	86	Manual and vHIT+	Biphasic Sequential	Bilateral Vestibular Neuritis Sequential	RH 0 30 LH 0 21	Two Acute events	50 dB loss High frequency
10	84	Manual HIT+ Chronic Skew	Biphasic Sequential	L AICA MCP stroke R vestibular Root entry stroke	Not Done	Two Acute events	High freq Hearing Loss R > L
11	58	Manual and vHIT+ Torsion swing Abnormal	Slowly Progressive BVL	Ataxia Saccade Hypermetria Abnormal pursuit Neuropathy SCA type 3 Cerebellar Atrophy	RH 0 22 LH 0 19 RA not done LA 0 09 RP 0 32 LP not done	No	Normal
12	49	Manual HIT Torsion swing Abnormal Calorics No ice response	Slowly progressive BVL	Idiopathic	Not done	No	
13	35	Manual and vHIT + Torsion swing Abnormal calorics: No ice response	Slowly Progressive BVL	Idiopathic	RH 0 14 LH 0 23 RA 0 38 RP 0 38 LA 0 35 LP 0 27	No	Normal
14	55	Manual and vHIT+ Torsion Swing Abnormal Calorics <5°/s SPV	Slowly Progressive BVL	Idiopathic	RH 0 72 LH 0 41 RA 0 71 RP 0 41 LA 0 42 LP 0 26	No	Normal
15	58	Manual and vHIT+	Slowly Progressive BVL	CANVAS Ataxia Neuropathy MRI Vermis atrophy	RH 0 29 LH 0 23 RA 0 22 LA 0 32 RP 047 LP 028	No	High frequency bilateral Hearing loss
16	43	Manual and vHIT+ Torsion swing Abnormal Caloric <5°/s SPV	Slowly Progressive BVL	Idiopathic	Not done	One episode	High frequency bilateral Hearing loss
17	79	T Manual and vHIT+ Torsion swing Abnormal Depressed SPV <5°/s	Slowly Progressive BVL	Idiopathic Hepatitis C	Not done	No	High frequency Hearing loss
18	82	Manual and vHIT+ Torsion Swing Abnormal Calorics depressed	Slowly Progressive BVL	Idiopathic	Nor done	Episodes for 5 years	High frequency Hearing loss
19		Manual and vHIT+ Audiogram	Slowly Progressive Cochleo-BVL	MELAS 3243 tRNA mutation Diabetes Retina Deg MRI Atrophy Calcifications	RH: 0.31 LH: 0.32 RP: 0.58 LP: 0.58 RA: 0.83 LA: 0.80	No	Deaf Unilateral Cochlear Implant
20		Manual and vHIT+ Audiogram Audiogram	Slowly Progressive Cochleo-BVL	MELAS 3243 tRNA Mutation Neuropathy	RH 0.31 LH 0.37 RP 0.58 LP 0.58 RA 0.83 LA 0,80	No	Severe Sensorineural loss Hearing loss. All frequencies
21	59	vHIT Calorics: No ice response	Slowly Progressive Cochleo-BVL	Superficial Siderosis	RH 0.08 LH 0.02 LA 0.17 RP 0.19 RA 0.25 LA 0.19	No	Severe 60 dB Sensorineural Hearing loss L > R
22	74	vHIT	Acute Lethargy	Right Meniere’s Intra-tympanic Streptomycin Left Idiopathic?	RH 0.29 LH 0.28 LA 0.26 RP 0.35 RA 0.26 LP 0.06	Yes	Deaf right ear Decreased Hearing left ear high frequency
23	68	Manual and vHIT+ Calorics Depressed	Episodic	R > L Vestibulopathy Sequential Neuritis	RH: 0.34 LH: 0.10	Yes	R > L sensorineural Hearing loss
24	59	Manual and vHIT+	Acute	SCA 3? Polycystic kidneys	RH: 0.58 LH: 0.60 LA 0.74 RP 0.64 RA 0.69 LP 1.03	No	Normal
25	66	Manual and vHIT+	Slowly Progressive	Anti-GAD Antibody +	RH 0.18 LH 0.32 LA 0.45 RA 0.45 LP 0.33 RP 0.33	No	Normal

**Figure 1:**
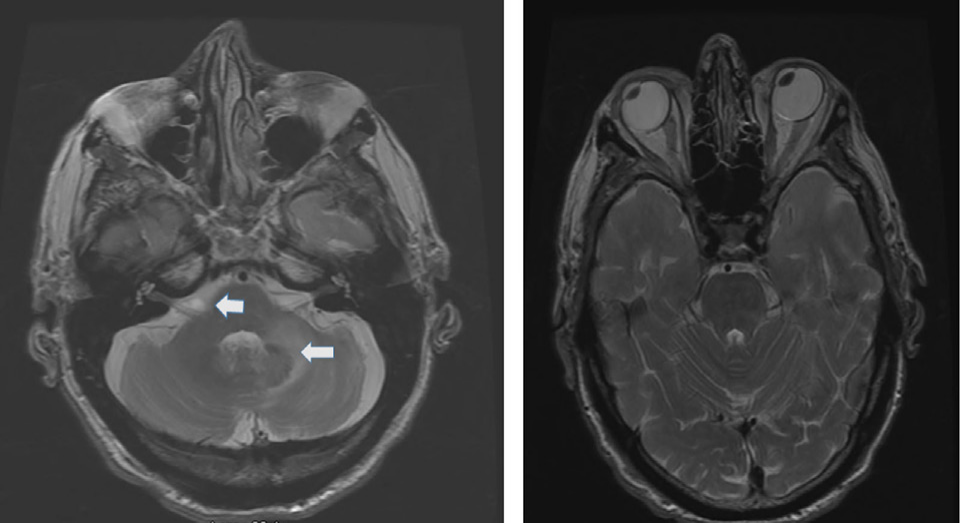
Axial T2 MRI: left panel shows a small area of increased signal intensity due to a small stroke involving the lateral pons, near the root entry of the right vestibular nerve (smaller arrow) and a larger stroke involving the left lateral pons and middle cerebellar peduncle (left panel larger arrow). Notice the conjugate ocular deviation of the eye to the right, coinciding with the slow phase of the nystagmus related to the second lacunar stroke in the right pons.

The etiology distribution (Tables 1 and 2) includes: idiopathic: 9, Wernicke’s encephalopathy: 11, superficial siderosis: 3, paraneoplastic syndrome: 3, bilateral vestibular neuritis (recurrent AVS lasting days without cochlear symptoms): 3, simultaneous ototoxicity of aminoglycoside and chemotherapy toxicity: 2, MELAS: 2, Meniere’s disease treated with intratympanic streptomycin in one ear: 1, acute phenytoin intoxication: 1, combined chronic unilateral tumor-related vestibulopathy and new contralateral vestibular neuritis (this patient presented with Betcherew’ s phenomenon): 1, bilateral AICA stroke: 1 (Figure 1), spinocerebellar ataxia type 3: second CANVAS: 2.

**Figure 2:**
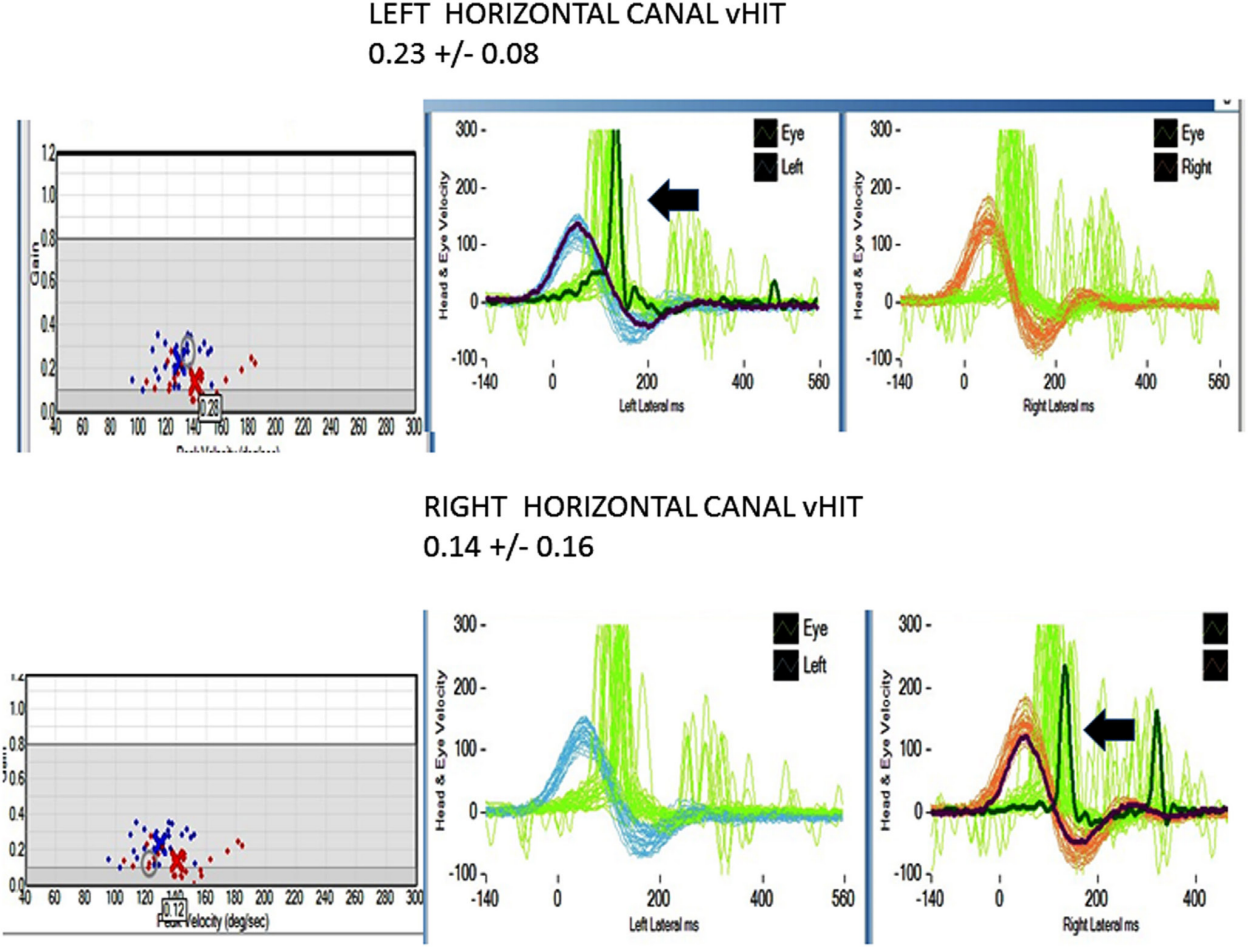
Horizontal video head impulse (h vHIT). Upper panel left h a-VOR gain is low 0.23 ± 0.08 (normal >0.7–1.0). The arrow points to a covert, corrective saccade, which was not apparent during the manual HIT. Lower panel right h a-VOR gain is low (0.14 ± 0.16). The arrow points to a covert, corrective saccade, which was not apparent during the manual HIT. The vertical canal gain was also low (not shown).

All patients underwent clinical testing as described in the protocol with the following result. Dynamic VA was abnormal in all patients. In five patients with primary gaze up (UBN) or downbeat (DBN), the nystagmus was a cofactor associated with impaired VA. We performed the manual HIT in all patients and recorded the vHIT in 30 patients examined when the vHIT device was first available to us. 39 patients had an abnormal manual HIT and two had covert saccades and low VOR gain (Figure 2). Prior to 2012, the patients underwent conventional bithermal caloric testing and 15 of them had absent caloric responses with conventional temperature stimulation, even when we utilized ice water stimulation. In few cases, standard caloric stimulation generated a weak horizontal nystagmus with slow phase velocities (SPV) of <5°/s. In some instances, the patients had caloric testing prior to our evaluation and we did not repeat it. Eight patients underwent torsion/swing chair testing decreased VOR gain and demonstrated phase lead when measured; the per-rotational and post-rotational nystagmus time constant was decreased (<5 s). Even though we did not routinely compare test of vestibular function in this series, we found them to be generally concordant in this small series (Figure 2).

In the patients with BVL due to Wernicke’s encephalopathy, the pretreatment seem thiamine levels were low in *n* = 8/11 cases, the remaining three had an infusion of high-dose intravenous thiamine without preceding serum level. Four of them had abnormal MRI findings typical for Wernicke’s encephalopathy.

## Discussion

In this cohort, BVL in *n* = 9/41 (~20%) was idiopathic as noted in previous reports (2–4). In addition, this series is biased toward BVL associated with a neurologic disease and thus differs from previous BVL series; therefore, the etiology frequency in our population must be interpreted with this fact in mind; however, we identified several BVL patients in the context of acute and subacute neurologic syndromes in *n* = 16/41 (>25%).

Recently developed diagnostic criteria provided precise clinical and laboratory signs of BVL (12). I utilized the proposed protocol in this retrospective series. Search for the BVL diagnostic gold standard test is a still matter of debate; because caloric testing, rotational testing, and manual or vHIT test lack 100% specificity/sensitivity, clinicians may use one or more of these modalities to confirm the clinically suspected diagnosis (3, 4, 15). However, the combination of head impulse test, dynamic VA, and Romberg testing while standing on a rubber foam cushion is a practical initial step in subacute and chronic to effectively select patients requiring additional investigation (16). I implemented further quantitative testing thereafter (4). In acute patients, the testing protocol varied and included dynamic VA and both manual and vHIT in all six semicircular canal (SCC) planes. I paid special attention to comparing the horizontal versus vertical canal VOR gain; in central, BVL lesions preferentially affecting the MVN and suggests thiamine deficiency as a potential cause (8, 10, 17), which may be confirmed with pretreatment serum thiamine levels and a favorable response to vitamin supplementation and normal diet (8–10, 17). Similarly, selective peripheral vestibulopathies may spare the anterior canal VOR (18). The value of testing the VOR gain in plane of all six-SCC cannot be overemphasized. I did not identify in this series, a single case of acute simultaneous, bilateral peripheral vestibulopathy.

Analysis of our findings suggest that an acute etiology is more frequent than anticipated. Neurologic patients now undergo routinely manual HIT and vHIT testing in acute in-patient units. The referral population served at our Center represents a mixed small urban and rural population; however, the referral base is large, probably in the order of three million patients. To facilitate the discussion will divide our cohort in acute and subacute bilateral vestibular loss (BVL) (symptom evolution <1 month duration) (Table 1) and chronic vestibular loss (Table 2) evolving over a period greater than 1 month, but usually of longer duration. In the chronic BVL group, several patients had either isolated bilateral peripheral loss, cochleovestibular loss, or mixed peripheral/central vestibular loss in the context of a neurodegenerative process.

### Acute BVL

Among the 41 cases, 16 patients had an acute/subacute presentation (11 Wernicke’s encephalopathy (WE), three paraneoplastic syndromes, and two additional cases included one with acute phenytoin intoxication and one acute right vestibular neuritis and with chronic posttumor resection-related contralateral vestibulopathy, who displayed Betcherew’s phenomena) (Table 1) (19). In WE, the presumed location of the lesion in WE is the medial vestibular nucleus (8, 17, 20); however, at present, temporal bone studies in WE have not been performed to my knowledge; therefore, it is unknown if a co-existent peripheral vestibulopathy is also present. None of our acute BVL patients had spontaneous primary gaze horizontal nystagmus, except for the one sequential patient with Betcherew’s phenomenon. *n* = 15/16 patients with acute/subacute bilateral vestibulopathy had direction changing, symmetric horizontal gaze evoked nystagmus (GEN). UBN in straight-ahead fixation was noted in five patients with acute thiamine deficiency (four with encephalopathy and one without) and in one PNS patient. UBN switched to chronic DBN in three cases and resolved in two. UBN was present in one PNS and DBN in a second PNS patient.

In the acute cases, and in concert with the subacute and chronic BVL cases, primary gaze horizontal nystagmus was absent. Development of bilateral symmetric or minimally asymmetric horizontal vestibular loss, regardless of acuity does not cause fixation horizontal nystagmus (21). To explain the horizontal GEN, failure of the horizontal neural integrator is likely responsible; this is the most common type of nystagmus present in Wernicke’s Encephalopathy (20) and other brainstem lesions (22, 23). Several acute patients had vertical nystagmus in primary gaze; however, the precise location of the lesion responsible is unknown. Candidate locations could be the medulla (nucleus intercalatus and nucleus of Roller) (17, 24, 25), the pons (superior vestibular nucleus) (26), or the interstitial nucleus of Cajal (27).

The overall outcome of the acute BVL patient was favorable. Among 11 thiamine, six deficient patients responded to thiamine replacement and a normal diet with complete recovery and three of them had normal h-VOR gain but developed chronic DBN and gait ataxia.

In addition, the acute phenytoin intoxication patient slowly normalized the VOR gain once the medication was properly titrated; the patient with Betcherew’s’ phenomenon improved spontaneously over a few days. Two of three paraneoplastic syndrome patients treated with immunosuppression, cancer specific management had continuous neurologic and medical deterioration, and died, and one of them declined treatment.

### Chronic BVL

The patients in this group comprised 25 patients (Table 2). Nine patients had isolated, idiopathic BVL, a number roughly comparable with previous BVL series (2–4, 28) Those patients with BVL due to ototoxic medications, together with those due to presumed sequential vestibular neuritis remain either clinically stationary, or are slowly improving through vestibular adaptation. One patient with bilateral sequential AICA strokes eventually had partial improvement after the second stroke (Figure 1); he was the only patient with chronic diplopia due to persistent, low-amplitude skew deviation.

In peripheral vestibulopathy, the manual HIT was abnormal in the plane of all canals tested, the vertical HIT in the plane of the posterior canal was, in our experience, easier to detect than the anterior canal, thus, often requiring vHIT for confirmation; however, the anterior canal VOR gain, as mentioned before, may be selectively spared in peripheral vestibulopathy (18). In previous series, exposure to ototoxic drugs is a frequent cause of BVL. Two patients in this series had BVL caused by exposure to gentamicin; curiously, both patients had gynecologic malignancies and had treatment with carboplatin and Taxol, they had discontinued all these medications when I first saw them. The lowest vHIT gain recorded in the series affected the ototoxic and idiopathic groups (near vestibular areflexia).

Patients with acute and those with chronic peripheral and central vestibular abnormalities had GEN, which was not present in isolated peripheral lesions. Eye movement abnormalities were frequent with central lesions (often diplopia due to extraocular muscle weakness, internuclear ophthalmoplegia, or skew deviation). We found additional eye movements abnormalities due to brainstem or cerebellar dysfunction. We did not test the VVOR routinely, thus, we cannot comment in its diagnostic value in this series; however, this would be another possible differentiating characteristic pointing to central localization and frequently present in CANVAS (29). In this series, the CANVAS patients had an abnormal VVOR (9). Sensorineural loss was more frequent with peripheral vestibulopathies.

A summary of the prognosis in this 41-patiet cohort includes Recovery in *n* = 14/41 cases, partial recovery, and progressive deterioration in *n* = 12/41. Two of the paraneoplastic syndromes died within 6 months after the diagnosis, because of cancer management complications and six patients are wheelchair bound (BVL with neurodegenerative disorders and two with superficial siderosis), the remaining 15 cases remain stable as noted in previous series (28).

In conclusion, the incidence and distribution of BVL varies with the population that attends the different centers and the specialty of the clinicians evaluating BVL interests in acute BVL is increasing because of portable technology and the possibility of BVL improvement. Table 3 is a summary of the main vestibular findings identified in the two groups of BVL patients.

**Table 3:** Comparative findings in bilateral vestibular loss.

Subacute/chronic bilateral peripheral vestibulopathy	Acute or subacute presumed central bilateral vestibulopathy
Impaired dynamic visual acuity	Impaired dynamic visual acuity
Abnormal manual and video head impulse (vHIT) horizontal head impulse test	Abnormal manual and vHIT horizontal head impulse test
Abnormal Manual vertical head impulse test The anterior canal gain may be selectively spared	Vertical manual and vHIT maybe normal or less affected than horizontal
Absent or depressed caloric responses	Absent or depressed caloric responses
No spontaneous horizontal fixation nystagmus Horizontal gaze evoked nystagmus (GEN) is generally not present (May have GEN or vertical nystagmus more commonly in CANVAS and SCA 3). In such cases, a combined peripheral and central vestibulopathy is present.	No spontaneous horizontal fixation nystagmus Horizontal GEN is present May have vertical nystagmus UBN in Wernicke’s, focal lesions of the brainstem, paraneoplastic syndrome DBN may be present in the chronic phase
Neurologic examination is usually normal, unless there is an associated neurodegenerative disorder	Neurologic examination is usually abnormal. Encephalopathy may be present
Sensorineural hearing loss may be frequently present, particularly in bilateral Meniere’s	Sensorineural hearing loss is usually not present
Imaging is usually normal, except when loss is associated with neurodegenerative disorder, superficial siderosis, and MELAS	Imaging is usually abnormal (acute signal changes in the gray matter surrounding the ventricles), cerebellar atrophy

## Ethics Statement

This study was approved by the University Of Illinois College Of Medicine IRB and follows the tenants of the declaration of Helsinski.

## Author Contributions

This is a single author paper. I examined each patient and conducted a retrospective review.

## Conflict of Interest Statement

Otometrics corporation loaned research equipment (beta site in 2012). This unit is no longer being used.
